# Two divergent chloroplast genome sequence clades captured in the domesticated rice gene pool may have significance for rice production

**DOI:** 10.1186/s12870-020-02689-6

**Published:** 2020-10-14

**Authors:** Ali Mohammad Moner, Agnelo Furtado, Robert J. Henry

**Affiliations:** 1 Genetic Engineering and Biotechnology Institute for Post Graduate Studies, University of Baghdad, Baghdad, Iraq; 2 Queensland Alliance for Agriculture and Food Innovation, University of Queensland, Brisbane, Qld 4072 Australia

**Keywords:** Rice, Chloroplast, Sequence, Domestication

## Abstract

**Background:**

The whole chloroplast genomes of 3018 rice genotypes were assembled from available sequence data by alignment with a reference rice chloroplast genome sequence, providing high quality chloroplast genomes for analysis of diversity on a much larger scale than in any previous plant study.

**Results:**

Updated annotation of the chloroplast genome identified 13 more tRNA genes and 30 more introns and defined the function of more of the genes. Domesticated rice had chloroplast genomes that were distinct from those in wild relatives. Analysis confirms an Australian chloroplast clade as a sister to the domesticated clade. All domesticated rice genotypes could be assigned to one of two main clades suggesting the domestication of two distinct maternal genome clades that diverged long before domestication. These clades were very distinct having 4 polymorphisms between all 1486 accession in clade A and all 1532 accessions in clade B. These would result in expression of 3 proteins with altered amino acid sequences and a tRNA with an altered sequence and may be associated with adaptive evolution of the two chloroplast types. Diversity within these pools may have been captured during domestication with subclades enriched in specific groups such as basmati, tropical japonica and temperate japonica. However the phylogenies of the chloroplast and nuclear genomes differed possibly due to modern rice breeding and reticulate evolution prior to domestication. Indica and aus genotypes were common in both chloroplast clades while japonica genotypes were more likely to be found in the same clade (cladeB).

**Conclusions:**

The different evolutionary paths of the cytoplasmic and nuclear genomes of rice have resulted in the presence of apparently functional chloroplast genome diversity and the implications for rice crop performance require further investigation.

## Background

Rice is crop of critical importance to global food security. Enhancement of genetic diversity is a key strategy for improving crop resilience to climate change and characterization of that diversity is important in managing food security. A wide range of rice types are grown throughout the world to satisfy the diverse food tastes of different human populations. Advances in genomics are now allowing large scale analysis of the entire genome. The 390 Mb nuclear genome of rice [1, 2] is responsible for the control of the traits that explain the differences in rice grain types. However, the 135Kb chloroplast genome is maternally inherited and more highly conserved making it a useful tool [3] for tracking maternal lineages in the domesticated gene pool and relating them to the diversity in wild populations. In rice, as in many other plant groups the maternal (chloroplast) and nuclear phylogenies may differ due to transfer of the maternal genome in rare cross pollination of plants from distinct populations.

Analysis of wild rices (*Oryza* species) in the group related to domesticated rice, the A genome species has revealed that the wild rices from South America and Africa (*Oryza longistaminata* and *O. glumaepatula*) have the most divergent chloroplast genome [4–6] while the Australian populations [7] (*O. meridionalis* [8]and related taxa) have the most divergent nuclear genomes [9]. The chloroplast genomes of wild *Oryza* have been widely studied and related to those of domesticated rice [4–6]. We recently reported a study of the whole chloroplast genomes of 58 mainly wild *Oryza*, identifying groups major groups based on geographic regions with some overlap in Asian accessions due to the absence of natural borders and human movement [5].

Recently DNA sequence data for more than 3018 domesticated rice genotypes was reported [10]. Reliable analysis of the whole genome sequence of the chloroplast was demonstrated for rice using sequence data from total plant DNA in rice [4]. Because of the high copy number of the chloroplast in the cell, reads from total plant DNA can be used to determine the whole chloroplast sequence with high accuracy. The pipeline used here (and explained in detail in the cited references) relies on the high abundance of the true chloroplast sequences in any NGS whole plant DNA analysis. The chloroplast is present in 1000 or more copies for every nuclear genome copy in most DNA preparations. Earlier studies of plant chloroplast genome sequences based upon PCR or cloning are all unreliable as these methods can preferentially amplify or clone copies of chloroplast genes in the nucleus or mitochondria. Here we use large numbers of high quality complete chloroplast genomes for the first time and avoid completely the risks of including nuclear or mitochondrial sequences. We now report analysis of the chloroplast genomes of the 3022 rice genotypes (Table 1) allowing a large-scale comparison of complete and accurate chloroplast genome sequences.

**Table 1:** Rices included in chloroplast genome analysis of 3091 genotypes

Sub clade (colour in Fig. 1)	Number of accessions/ clade	Percentage of genotypes
Australian (red)	33	1.07
Wild Asian (green)	31	1
Clade A (purple)	1486 (1203 subsp. Indica)	48.1 (81% of this clade is indica)
Clade B (blue)	1532 (538 subsp. Indica)	49.6 (35% of this clade is indica)
African	5	0.16
South American	3	0.1
Out group	1	0.03
Total	3091	100

## Results

**Fig. 1:**
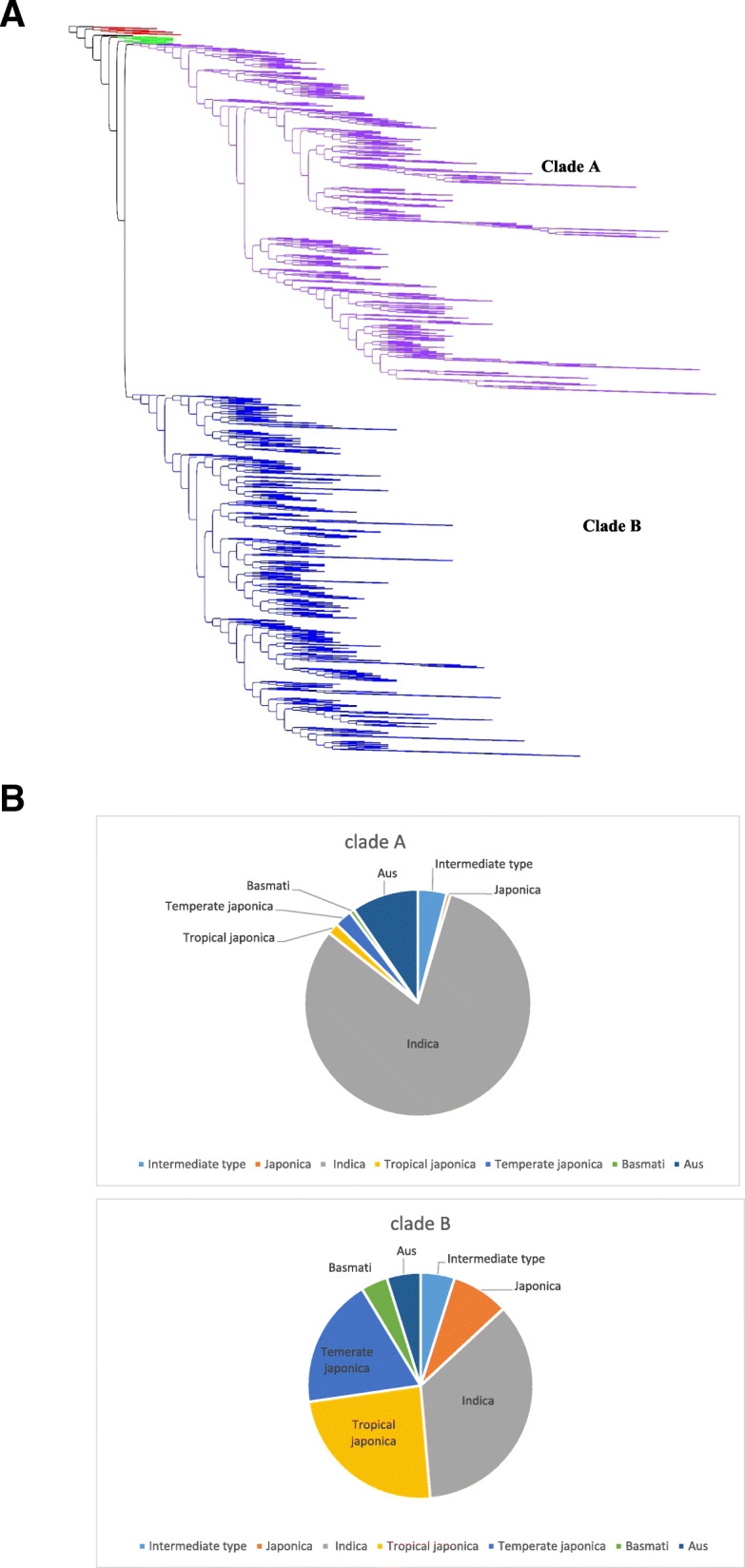
**a** Relationship between rice chloroplast genomes Australian (red), wild Asian (green), domesticated rice Clade A (purple), domesticated rice Clade B (blue), *Oryza officinalis* (outgroup). **b** sub species percentages in each main clade

The analysis of the 3091 rice chloroplast genomes allowed analysis of the relationships within the domesticated rice gene pool and with the main groups of wild relatives. A reliable whole genome sequence was generated from the total DNA sequence avoiding the complications of errors due to chloroplast insertions in the nucleus or mitochondria that have plagued earlier chloroplast studies [3]. The chloroplast genomes of domesticated rices were distinct from those of the wild rice relatives included in the analysis (Fig. 1a and b). The Asian wild rices included in this study were a sister clade to the domesticated rices and the Australian wild rices a sister to all the Asian wild and domesticated rices. The domesticated rices were separated into two main clades. Clade A and Clade B contained a range of rice types (Table 2). The main rice types, japonica (including tropical japonica and temperate japonica, indica, basmati and aus were all found in both clades but in different proportions (Table 2). African rice, *O. glaberrima* and wild African rice, *O. bathii,* were found within Clade A (Fig. 1).

**Table 2:** Distribution of rice types between 2 major chloroplast clades

Type or sub-species	Number of accessions	% of total accessions	Number clade A	Number clade B
Intermediate type	135	4.5	61	74
Japonica	132	4.4	8	124
Indica	1741	57.7	1203	538
Tropical japonica	388	12.9	24	364
Temperate japonica	319	10.6	37	282
Basmati	68	2.3	10	58
Aus	215	7.1	142	73

**Fig. 2:**
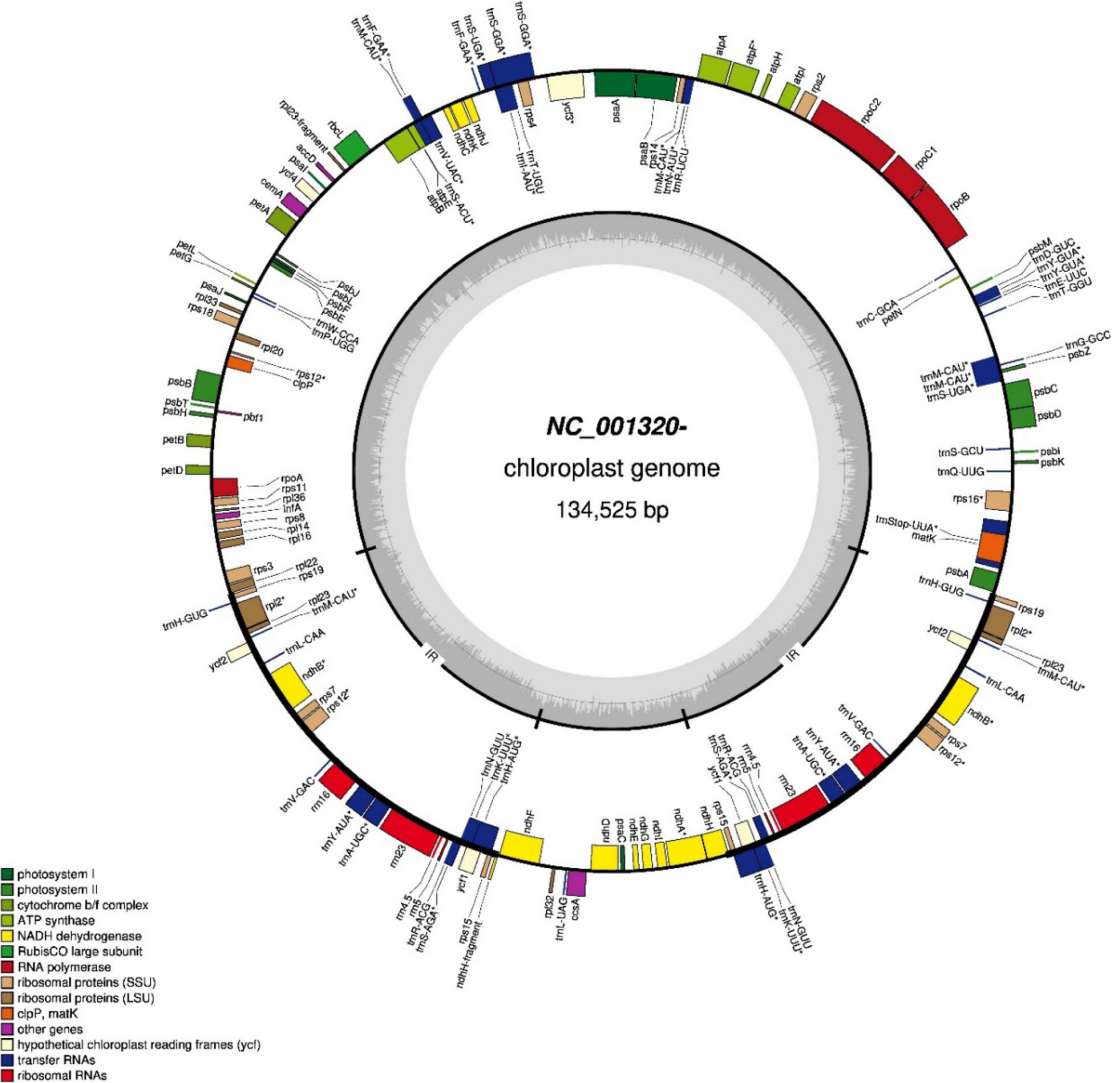
Annotated rice (*O. sativa* Nipponbare, NC_001320), chloroplast genome annotation with GeSeq provide a significant update (supplementary data File 1 and File 2) to the previously reported annotations, the grey circle represents the GC content and defines the main chloroplast fragments LSC, SSC, IRA an

The chloroplast genome was annotated using newer analysis tools to produce a more complete annotation (Fig. 2) as has recently been developed for other plant species [11]. Annotation with GeSeq [12] rather than DOGMA as used in earlier rice chloroplast genome annotations revealed 51 rather than 38 tRNA genes, 31 rather than 1 intron and provided a gene identity for all proposed genes while the earlier method indicated many genes without identity including” hypothetical proteins” (supplementary data files 1 and 2).

The two clades had sequences that suggested functionally different chloroplasts. All members of clade A differed from all members of clade B in 4 positions that changed the encoded amino acid sequence of the encoded protein or sequence of a tRNA (Table 3). The RNA polymerase (rpoC2) with 2 changes, ribosomal protein (rpl20) and a tRNA were polymorphic between the two clades. The photosynthesis gene, psb B was also polymorphic but unlike the others this polymorphism did not strictly associate with the two clades. In clade A 88 were C and 1398 were T and in clade B there were all C (1532) and no T.

**Table 3:** Sequence polymorphisms between chloroplast clades. Positions involving polymorphisms associated with amino acid changes are in bold

Position in chloroplast genome	Polymorphism between clades B/A	Gene	Amino acid change or predicted SNP impact (Genescan)
412	T /C	psbA	no
4547	G /T	rps16 exon 2	no
6282	T/C	–	
6608	G/T	–	
7134	T/C	psbK	no
8127	A/G	–	
8599	G/A Mixed	–	
12496	A/G	–	
12799	G/A	Intron trnM-CAU	
12819–12820	TA/CC	–	
13468	A/T	–	
14231	A/G	trnl	RNA
15908	T/A Mixed	intron trnY-GUA gene	
17203	A/G	–	
17746–17747	GG/AA	–	
18479	T/G	–	
18520	G/A	–	
20587	T/A	rpoB	no
**28019**	G/T	rpoC2	Tryptophan to leucine W to L
**29113**	A/G	rpoC2	Asparagine to aspartic acid N to D
35382	G/A	atpA	no
49856	C/T	–	
50250	A/C	–	
51349	T/A Mixed	intron trnS-ACU gene	
52147	C/T Mixed	atpB	no
53521	C/T	–	
55788	G/T	–	
56588	T/C	Acetyl-CoA	no
56861	C/T	Acetyl-CoA	no
60995	T/G	–	
64166	C/A	–	
**66402**	A/G	rpl20	Serine to proline S to P
**69349**	C/T Mixed	psbB	Alanine to valine A to V
74268	A/C Mixed	–	
77793	G/A	rpl16 exon 2	no
93081–93083	AAA/TTT	–	
102927	C/T	ndhF	no
104286	G/A	–	
106348	G/T	–	
108269	G/A	–	
108662	C/	–	
109142	A/G	–	
113077	G/T	ndhH	no
122035	TTT/AAA	–	
125723	A-G Very Mixed	–	
125779	G-T Very Mixed	–	
125800	G-A Very Mixed	–	

For more details of the two clades see supplementary file 3.

## Discussion

Earlier *Oryza* chloroplast phylogenies examined the diversity of chloroplasts from divergent species across the genus [6]. The range of wild rice chloroplast genome sequences in samples collected in Asia may be due to both divergence in the wild and gene flow from domesticated to wild populations making interpretation of the basis of relationships difficult. Rices with chloroplasts very similar to those in the domesticated gene pool may be found in Asian collections [5] but the direction of gene flow may be difficult to establish without detailed populations studies as revealed in a recent study of wild and domesticated barley [13]. The current study expands specifically on the relationships within the clade that includes Asian domesticated rices. The current phylogeny includes far more samples than any previous study (more than 3000) and is likely to be more accurate [14] than any earlier study. Dramatically larger numbers of domesticated and more wild rices are now included in the analysis.

Recent analysis of the diversity of domesticated rice based upon nuclear genome analysis suggests three separate domestication of the japonica, indica and aus type [15] based upon nuclear genome analysis.

The extensive analysis of domesticated rices in this study does not refute this but shows that domesticated rice genotypes can also all be grouped into two main clades strongly suggestive of domestication of two distinct clades of the cytoplasmic genome supporting the concept of multiple origins contributing to a single domestication [16]. These two clades (A and B) may be in part associated with the distinct origins of japonica and indica rice. However, the two chloroplast clades generated by neighbour joining and subsequently distinguished by unique polymorphisms (Table 3) each contained a wide range of rice types (Table 2). Clade A included mainly indica rice. Most japonica rices were in clade B with distinct chloroplast sub-clades for basmati, tropical japonica and temperate japonica. Despite these general groupings most groups were mixed. Indica rices were predominant in clade A but also widely distributed across clade B including all sub-clades. Clade A had more aus types but many were also present in clade B, confirming the earlier report of aus rice types with both main types of chloroplast [5]. These results demonstrate that cytoplasmic genomes and nuclear genomes have been widely recombined to generate the current domesticated rice gene pool. Rice types are likely to be overwhelmingly determined by the nuclear genome but the chloroplast genome may often have originated from another rice type. The extent to which this is a product of genetic recombination in the breeding of modern varieties or due to events of domestication or following domestication is not clear.

Domesticated rice appears to have common domestication loci [15] but be derived from more than one ancestral population. One model for rice domestication suggests domestication of japonica and possibly indica from distinct wild populations corresponding to *O. rufipogon* and *O. nivara* respectively. This needs to be followed by some level of introgression to generate the two distinct types of cultivated rice with common alleles at domestication loci. The crosses between these two populations could have either parent as the maternal parent allowing domesticated populations with both chloroplast types to evolve. This potential is illustrated by recent evidence for wild populations generating hybrids by crossing in both directions [17]. The existence of reproductive barriers [17] may result in unidirectional pollen flow and rapid nuclear genome replacement or chloroplast capture. The implications of the chloroplast genome type for plant performance are not known but may be significant.

Analysis of the domestication of crop plants is complicated by the potential for wild populations to originate from domesticated plants that develop traits that allow them to perform well outside cultivation. Gene flow from domesticated to wild populations can also further complicate analysis. Early domesticates like rice may have had a longer period over which these processes may have occurred. Barley, possibly the first plant domesticated, was domesticated in the fertile-crescent. The presence of distinct wild barley populations in Tibet suggested the possibility of a second independent domestication. However, recent genome analysis showed that the wild barley in Tibet may have been derived from the domesticated barley introduced to the region by humans in the last 4000 years [13].

In the case of rice evidence for close relationships between wild and domesticated rice has been used as evidence for the primary site of domestication [18]. However, gene flow from domesticated to wild populations may also explain a close relationship. The two distinct chloroplast genomes found in the *Oryza sativa* gene pool is only easily explained by the domestication of two distinct maternal genomes. The nuclear genome domestication history is more difficult to define but the two main types, indica and japonica, are likely products of separate domestications. Human movement of rice and modern plant breeding have resulted in the widespread recombination of nuclear and maternal genomes reported here. Domestication loci may now be shared across the modern *O. sativa* gene pool despite at least two separate primary domestications that have capture two distinct maternal gene pools. Indica and japonica types have long been recognized [1]. A third group, aus, has been explained by a third possible domestication. The chloroplast evidence presented here shows that aus genotypes all have one of the two main types of chloroplast genome and do not represent a separate maternal domestication.

The chloroplast and nuclear genome phylogenies show significant discordance across the *Oryza*.

*O. longistaminata* in Africa and *O. glumaepatula* in South America have similar chloroplast genomes [4] despite their more distant nuclear genome sequences. *O. glumaepatula* is a part of the A genome clade of close relatives of domesticated rice but has a divergent chloroplast genome being the most divergent A genome species. However nuclear genome analysis puts the diverse *O. meridionalis* [19, 20] from Australia as the most divergent in the A genome clade [9].

Another example is provided by the Australian populations with morphological similarity to *O. rufipogon* in Asia. This taxa has a chloroplast genome that is close to that of wild rices in Australia [21] but a nuclear genome that groups it with the Asian wild rices [9]. The general conclusion from this analysis is that chloroplast transfer between closely related *Oryza* taxa has been widespread. The recent discovery [22] of some wild populations of hybrids between taxa indicates that this is an ongoing evolutionary process in the *Oryza*.

The two maternal lineages found in domesticated rice are very distinct (Table 3) suggesting a long period of divergence of these two ancestral types. The presence of functional polymorphisms that alter the encoded amino acid is significant especially given the highly conserved nature of the chloroplast genome. Divergence times of the order of more than half a million years have been suggested for these chloroplasts [6]. The annotation tool, Geseq, allowed the detection of more genes than earlier tools that have been widely used for chloroplast annotation. Some genes of unknown function (hypothetical proteins) are now correctly annotated (Supplementary files 1 and 2). Additional tRNA genes were revealed in the chloroplast genome. Geseq has significant advantages as it uses more de novo predictors and searches by profile hidden Markov model (HMM) in comparison to the classic annotation procedure. In addition, this tool has identified more introns in chloroplast genes [12].{Tillich, 2017 #2} reference 13.

Functional adaptation of *Oryza* chloroplasts has been suggested. The photosynthesis gene psb B that was found in this study to be polymorphic in domesticated rice has been implicated in adaptation to shade and sun in *Oryza* species growing in different habitats [23]. The other chloroplast genes that show differences that would result in changes in protein sequence are associated with gene expression; being 2 changes in an RNA polymerase gene (rpoC2) and a difference in a ribosomal protein (rpl20). The two chloroplast clades also show a consistent difference in a tRNA sequence. The presence of an alanine/valine polymorphism in the psb B gene suggests the possibility of parallel adaptation in the two chloroplast lineages. The impact of these differences on rice performance in different environments is worth careful evaluation as may be the use of chloroplast genomes from wild relatives [24]. The potential for genetic improvement of rice by selection of the maternal genome requires careful analysis. This would be facilitated by the availability of rice genotypes with near identical nuclear genomes and divergent maternal genomes.

## Conclusions

This study has demonstrated that rice genetics is not a simple dichotomy with indica and japonica domestications but rather two domestications of distinct maternal genomes and introgression of many genes from wild populations. This suggests that domesticated rice has many “fathers” but only two “mothers (or groups of mothers)”.

The evolution of the *Oryza* genus [25] demonstrates patterns of divergence, introgression and natural and human selection [26] that have allowed both the cytoplasmic and nuclear genomes to evolve with some independence especially for closely related taxa for which chloroplast transfer is possible. The rich data set provided by the sequences of the 3091 genotypes [10] analysed here has proven to be an important resource for rice biology and has been used successfully in other studies [27].

Rice breeding has not focused on selection of maternal genomes. This study suggests that the analysis of the performance of different chloroplast genomes (the two types in domesticated rice and others from wild populations) with a common nuclear genome may be necessary to define the potential for chloroplast genome selection to advance rice performance in different environments. The maternal genome may influence traits influencing adaptation to specific environments and this may determine crop performance in specific regions. Given the importance of rice as a food crop this possibility requires careful evaluation.

## Methods

Next generation sequence data of 3018 domesticated rice varieties, was obtained from the NCBI database [28]. Methods for chloroplast genome sequencing and assembly have been detailed in earlier studies [5]. A detailed discussion of chloroplast genome sequencing and assembly is provided by Moner et al. (2018) [5]. Complete chloroplast genome sequences were obtained by alignment with a rice chloroplast reference genome sequence. Sequence assembly by mapping was used as the only practical approach for the large number of closely related (domesticated) genomes while in earlier studies with much very more diverse genomes mapping and de novo based approaches have been reconciled manually to generate the genome sequence. Raw reads sequence were subjected to quality checking and trimmed to obtain high quality reads, trimmed reads were mapped to the chloroplast reference sequence NC_001320.1 using bwa-mem version 0.7.13 [29] (FastQC tool with *fastqc -o* for overall QC and then *cutadapt -m X -q 20* for trimming) and refined by further mapping rounds using GATK version 3.6 [30] Reads were aligned to the Nipponbare reference sequence using BWA-MEM (release 0.7.10). The reads around indels were realigned for phylogenetic analysis using GATK RealignerTargetCreator and IndelRealigner package (release 3.2–2) [9, 22].

The chloroplast mapping data for the 3091 samples had a sequencing depth of between 55 X (lowest sample with 88,478 reads of 100 bp) and 10,863 X (highest sample with 17,382,581 reads of 100 bp), to ensure an accurate calling of the consensus sequence of the chloroplast for each sample. The average coverage for all samples was 2215 X.

Australian and Asian wild rice chloroplast sequences were included and a sequence from Oryza officinalis was used as an out group (Table 1). Chloroplast sequences were aligned using MAFFT [31] utility with the following setting (Auto, 1PAM/K = 2 scoring matrix, 1.53 open gap penalty and 0.123 offset value). In order to perform phylogenetic analysis, two strategies were used: all chloroplast sequences, the wild relatives and out group were aligned together, and secondly they were divided into groups according to their sub species regardless of the country of origin. Phylogenetic tree construction was performed using Geneious V 9.1 using the Neighbour –Joining method with the Tamura-Nei genetic distance model. Fasta sequences of the annotated chloroplast sequence of *O. sativa* Nipponbare (NC_001320), were analysed using the web tool GeSeq to annotate plain sequence using default parameters: protein search identity =25, rRNA, tRNA, DNA search identity =85. The utility of this tool in generating high quality chloroplast annotations has recently been demonstrated. They were then uploaded to OGDRAW to visualise the chloroplast genome. The annotated file was imported into Geneious to compare with the original annotated file. Differences were evaluated manually with gene by gene comparison of the annotation tables.

## Supplementary information

**Additional file 1.** (CSV 14 kb)

**Additional file 2.** (CSV 17 kb)

**Additional file 3.**

## Data Availability

All original data is available from the NCBI database [1]. The chloroplast genome datasets generated during the present study are available at the follow site: DOI 10.6084/m9.figshare.13040462
