# Supplementary figures and images for: Two divergent chloroplast genome sequence clades captured in the domesticated rice gene pool may have significance for rice production
Source: BMC Plant Biol. 2020 Oct 14;20:472. doi: 10.1186/s12870-020-02689-6 (PMC7558744; doi:10.1186/s12870-020-02689-6)

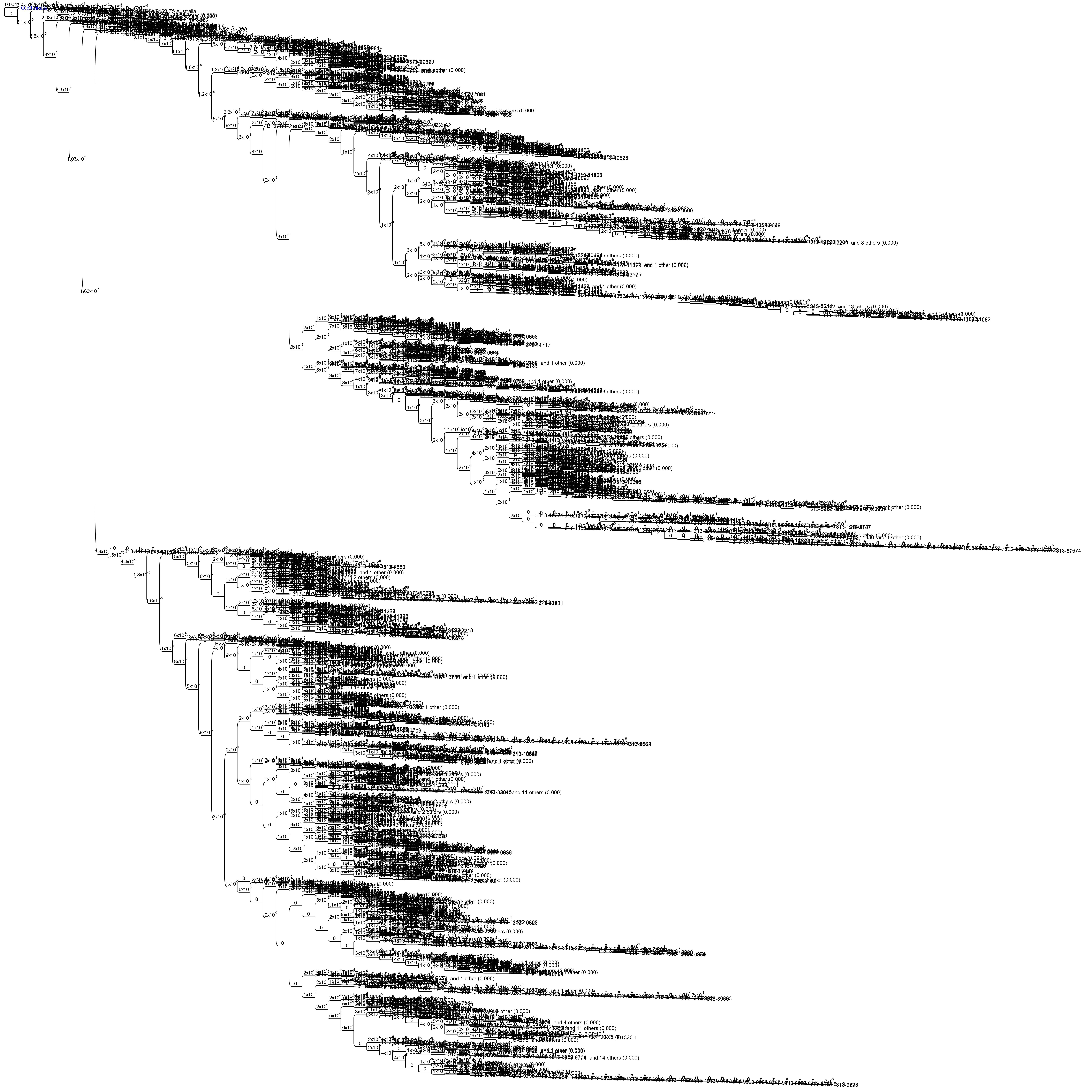

Supplement: Supplementary file 3 — Additional file 3. [file 12870_2020_2689_MOESM3_ESM.jpg]
